# The Anaesthetics Isoflurane and Xenon Reverse the Synaptotoxic Effects of Aβ_1–42_ on Megf10-Dependent Astrocytic Synapse Elimination and Spine Density in Ex Vivo Hippocampal Brain Slices

**DOI:** 10.3390/ijms24020912

**Published:** 2023-01-04

**Authors:** Dai Shi, Jaime K. Y. Wong, Kaichuan Zhu, Peter G. Noakes, Gerhard Rammes

**Affiliations:** 1 Department of Anesthesiology and Intensive Care, Klinikum Rechts der Isar, Ismaningerstraße 22, 81675 Munich, Germany; 2 School of Biomedical Sciences, The University of Queensland, St. Lucia, QLD 4072, Australia; 3 German Center for Neurodegenerative Diseases, Feodor-Lynen-Straße 23, 81377 Munich, Germany; 4 Center for Neuropathology and Prion Research, Feodor-Lynen-Straße 23, 81377 Munich, Germany; 5 Queensland Brain Institute, The University of Queensland, St. Lucia, QLD 4072, Australia

**Keywords:** Alzheimer’s disease, dendritic spine density, MEGF10, astrocytes, synapse elimination, phagocytosis

## Abstract

It has been hypothesised that inhalational anaesthetics such as isoflurane (Iso) may trigger the pathogenesis of Alzheimer’s disease (AD), while the gaseous anaesthetic xenon (Xe) exhibits many features of a putative neuroprotective agent. Loss of synapses is regarded as one key cause of dementia in AD. Multiple EGF-like domains 10 (MEGF10) is one of the phagocytic receptors which assists the elimination of synapses by astrocytes. Here, we investigated how β-amyloid peptide 1–42 (Aβ_1–42_), Iso and Xe interact with MEGF10-dependent synapse elimination. Murine cultured astrocytes as well as cortical and hippocampal ex vivo brain slices were treated with either Aβ_1–42_, Iso or Xe and the combination of Aβ_1–42_ with either Iso or Xe. We quantified MEGF10 expression in astrocytes and dendritic spine density (DSD) in slices. In brain slices of wild type and AAV-induced MEGF10 knock-down mice, antibodies against astrocytes (GFAP), pre- (synaptophysin) and postsynaptic (PSD95) components were used for co-localization analyses by means of immunofluorescence-imaging and 3D rendering techniques. Aβ_1–42_ elevated pre- and postsynaptic components inside astrocytes and decreased DSD. The combined application with either Iso or Xe reversed these effects. In the presence of Aβ_1–42_ both anaesthetics decreased MEGF10 expression. AAV-induced knock-down of MEGF10 reduced the pre- and postsynaptic marker inside astrocytes. The presented data suggest Iso and Xe are able to reverse the Aβ_1–42_-induced enhancement of synaptic elimination in ex vivo hippocampal brain slices, presumably through MEGF10 downregulation.

## 1. Introduction

Alzheimer’s disease (AD) is the most common cause of dementia in elderly adults [1]. The neurodegeneration seen in AD involves synaptic elimination, loss of neuropil, neurotransmitter disturbances and accumulation of extracellular β-amyloid (Aβ) deposits [2,3,4]. Although the cause of AD is not fully understood, soluble Aβ-oligomers have been reported to be more toxic to neurons and induce more synapse loss than extracellular plaques [5,6,7,8,9]. Previous studies revealed that the loss of synaptic contacts might be the physical basis for dementia in AD [10,11,12]. As the main pathogenic form of Aβ, Aβ_1–42_ has been reported to be related to synapse loss, and responsible for the decrease of dendritic spine density (DSD) [13]. 

The dentate gyrus, as the beginning of the hippocampal circuit, is considered to be vital for associative memory [14]. Previous studies reported that there was a 35–44% reduction of spine number and a 60% reduction of spine density in the dentate gyrus in AD [15,16]. In the early stage of AD, the loss of neuro-connectivity in dentate gyrus was considered to promote memory decline [17]. 

In clinics, the elderly patients usually have higher prevalence of postoperative cognitive dysfunction (POCD) than the young patients [18,19]. The commonly used inhalational anaesthetic Iso may accelerate the pathogenesis of AD and lead to cognitive decline in elderly adults [20,21,22,23]. As a general anaesthetic, Xe is well established as a neuroprotective agent [24,25,26], but still cannot reduce the incidence of POCD in patients compared to other anaesthetics [19]. It has been reported that Xe, as a low potent NMDA receptor antagonist, could reversibly attenuate long-term potentiation (LTP) in the hippocampus [27], implying a potential effect on the plasticity of synapses [28]. However, it is still unclear whether Iso and Xe have influence on the synapse loss during the progression of AD.

As the main glial cell type in the brain, astrocytes were established to have a close link with AD and aging and highlighted the important role in the controlling of synapse elimination in the CNS [29]. It was reported that astrocytes assist in the elimination of synapses via phagocytic mechanisms utilizing the Multiple EGF-like domains 10 (MEGF10) pathway [30,31]. MEGF10 a phagocytic receptor present on astrocytes, recognizes the opsonin C1q [32]. C1q as an opsonin forms a ‘eat me signal’ via its binding to exposed phosphatidylserine or desidylated proteins on the surfaces of stressed neurons (e.g., pre and postsynaptic elements) [32,33,34]. Astrocytes displayed approximately 50% reduction in the relative engulfment ability when MEGF10 was blocked, suggesting that MEGF10 is one of the major phagocytic receptors that astrocytes use to eliminate synapses [30,35,36,37].

In order to explore the association between inhalational anaesthetics and aetiology of AD, we investigated the effect of Iso and Xe on the synapse elimination by astrocytes, and the role of MEGF10 in this elimination in the presence of Aβ_1–42_ in this study.

## 2. Results

### 2.1. The Effect of Iso or Xe on DSD

**Figure 1:**
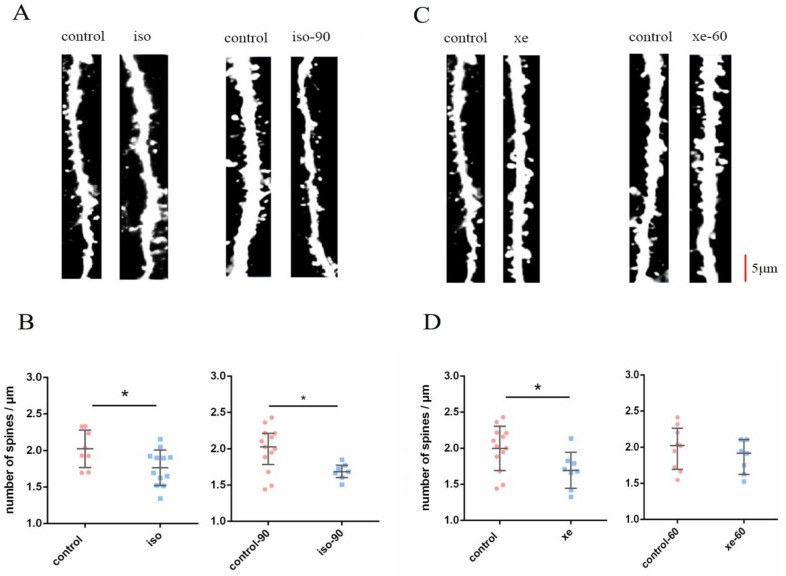
(**A**,**C**) show representative dendrites from the control group and the isoflurane (Iso)/xenon (Xe) treated groups, at treatment and post wash out (60/90 min) of gas, respectively. Scale bar = 5 μm. Dendrites were analyzed from brain slices of GFP-M mice. (**B**,**D**) left graphs show both Iso and Xe reduced the DSD in the dentate gyrus of hippocampus (*p* = 0.0201 for iso, and *p* = 0.0244 for Xe, Mann–Whitney U test,). (**B**,**D**) right graphs show the reduction of DSD caused by Xe was reversed (*p* = 0.3441 Mann–Whitney U test), but not for Iso (*p* = 0.0292, Mann–Whitney U test). Error bars in all graphs indicate the means +/− SDs. Every data point in the scatter plots represents the mean spine density from 8–10 dendrites per one animal (*n* = 8–13). Dendrites that were 15 µm away from their respective cell’s soma were chosen for these analyses. Black horizontal bars below the * indicate a significant difference (*p* < 0.05) between the groups.

We investigated the effect of Iso and Xe alone on the DSD. Compared to the control group, DSD decreased after the treatment with either 0.37 mM Iso or 1.9 mM Xe. Post removal of Iso, DSD remained evident up to 90 min, whereas the effect of Xe on DSD was reversed 60 min after Xe removal (Figure 1). Together these results suggest both of the anaesthetics influence the DSD while Xe has a weaker effect.

### 2.2. The Effect of Aβ_1–42_ Combined with Iso and Xe on DSD

**Figure 2:**
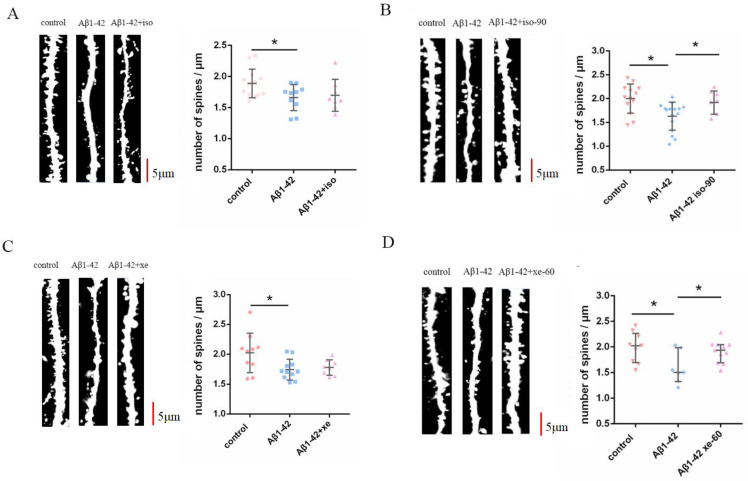
The combined application with either Iso or Xe reversed the Aβ_1–42_-induced DSD attenuation. (**A left**)—representative dendrites for control, Aβ_1–42_, alone and the Aβ_1–42_ combined with Iso group. (**A right**)—the DSD of the Aβ_1–42_ combined with Iso group did not restore immediately after Iso treatment. (**B left**)—representative dendrites for control, Aβ_1–42_ alone and Aβ_1–42_ combined with Iso plus 90 min washout. (**B right**)—after 90 min Iso washout, DSD recovered to control, *t*-test, * *p* = 0.0284. (**C left**)—representative dendrites for control, Aβ_1–42_, and Aβ_1–42_ combined with Xe. (**C right**)—DSD in the presence of Aβ_1–42_ combined with Xe was not affected immediately after Xe treatment. (**D left**)—representative dendrites for control, Aβ_1–42_ alone, and Aβ_1–42_ combined with Xe plus 60 min washout. (**D right**)—after 60 min of Xe washout, DSD recovered to control. Mann–Whitney U test, * *p* = 0.0266. Scale bars = 5 μm. Error bars in all graphs indicate the means +/− SDs. Every data point in the scatter plots represents the mean spine density from 8–10 per one animal (n = 8–13). The dendrites are from brain slices of the GFP-M(Thy1-eGFP) mice. Dendrites that were 15 µm away from their respective cell’s soma were chosen for these analyses. Black horizontal bars below the ∗ indicate a significant difference (*p* < 0.05) between the groups.

Given that both Iso and Xe can reduce DSD, we sought to determine if the added presence of Aβ_1–42_ a known inducer of DSD, had additional effects on DSD. We measured DSD after treating brain slices of GFP-M mice with 50 nM Aβ_1–42_. Compared to the control group Aβ_1–42_ decreased DSD significantly (Figure 2).

The Aβ_1–42_ pre-treated brain slices were gassed with 0.37 mM Iso for 90 min or 1.9 mM Xe for 60 min. We tested DSD immediately after the treatment with anaesthetics as well as 90 min and 60 min from the time point of removing Iso and Xe, respectively. The Aβ_1–42_ -induced decrease in DSD was not affected immediately after the treatment with either anaesthetic (Figure 2A,C), whereas the reduction of DSD caused by Aβ_1–42_ recovered after 60/90 min wash-out of the respective anaesthetic (Figure 2B,D). These results suggest that Aβ_1–42_ induced a reduction of DSD, while the combined application with either Iso or Xe reversed this spine attenuation 90/60 min after anaesthetics have been removed.

### 2.3. The Effect of Aβ_1–42_ on Astrocyte-Dependent Elimination of Synapses Could Be Reversed by Iso or Xe

**Figure 3:**
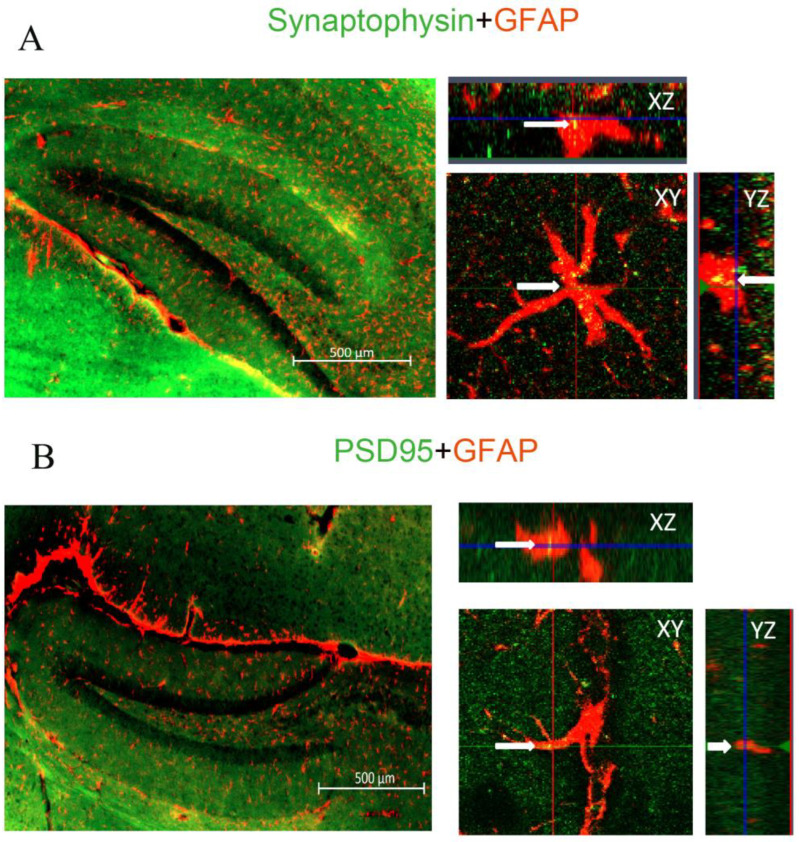
Pre- and postsynaptic material was engulfed by astrocytes in hippocampal slices. (**A**) shows the presynaptic marker synaptophysin (green) localize inside the astrocyte (red), indicated by the yellow—merge of green and red. The arrow points the synaptophysin inside the astrocyte in the XY, YZ, XY planes of view (yellow). (**B**) shows the postsynaptic marker PSD95 (green) localize (yellow) inside the astrocyte (red). The arrow points to the PSD95 inside the astrocyte in the XY, YZ, XY planes of view (yellow). These microscopic images reveal that both pre- and postsynaptic elements are inside astrocytes, which suggests that these synaptic components have been phagocytosed by astrocytes. Scale bar of the right panel = 10 μm. The images are from the brain slices of C57BL/6 mice.

**Figure 4:**
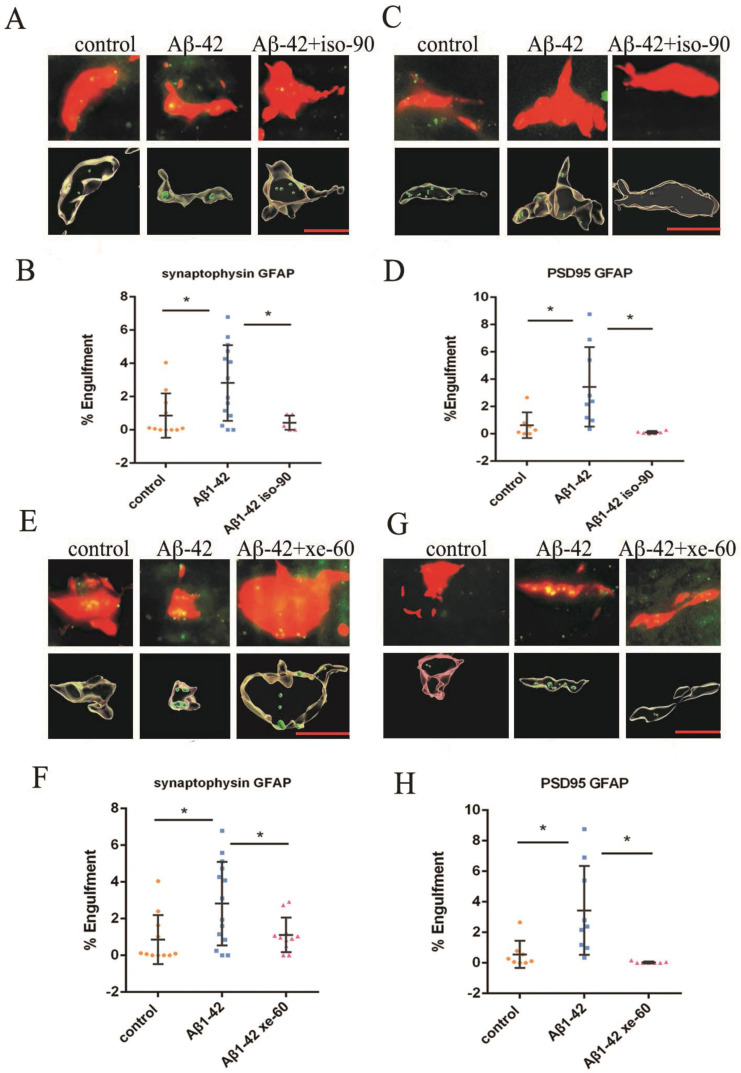
Aβ_1–42_ elevated pre- and postsynaptic marker inside astrocytes. Anaesthetics restore Aβ_1–42_-induced elevation of astrocyte-dependent synaptic engulfment in hippocampal slices. (**A**,**E**) the red, green and yellow colours represent the astrocyte (GFAP, red), presynaptic marker (synaptophysin, green) and merge of GFAP and synaptophysin (yellow), respectively (top panels). The lower panels in (**A**,**E**) represent the same cell but rendered into 3D using IMARIS software (Bitplane). The raw image data (apotome and respective 3D IMARIS reconstructions) for (**A**,**E**) are seen Supplementary Figures S1A and S2A, respectively). (**B**,**F**) the results show an increase of synaptophysin stained material inside the astrocyte when treated with Aβ_1–42_ (% of engulfment, Mann–Whitney U test, * *p* = 0.0194). (**C**,**G**) the red, green and yellow colours represent the astrocyte (GFAP, red), postsynaptic marker (PSD95, green) and merge of GFAP and PSD95 (yellow), respectively. The lower panels in (**C**,**G**) represent the same cell but rendered into 3D using IMARIS software. The raw image data (apotome and respective 3D IMARIS reconstructions) for (**C**,**G**) are seen Supplementary Figures S1B and S2B, respectively. (**D**,**H**) Aβ_1–42_ elevated postsynaptic marker inside astrocytes (% of engulfment, Mann–Whitney U test, * *p* = 0.0078). (**B**,**D**,**F**,**H**) the dot plots showed that the combined application with either Iso or Xe reversed elevation of synaptic markers inside astrocytes (B, F, % of engulfment, Mann–Whitney U test/*t*-test, * *p* = 0.0241/0.0292. (**D**,**H**), % of engulfment, Mann–Whitney U test, * *p* = 0.0004/0.0002). Scale bar = 5 μm. Error bars in all graphs indicate the means +/− SDs. Every data point in the scatter blot represents one animal (*n* = 4–10). The images are from the brain slices of C57BL/6 mice, The % of engulfment of either pre- (synaptophysin) or post- (PSD95) synaptic material in (**B**,**D**,**F**,**H**) within astrocytes were assessed by employing the methods of Schafer et al., to our 3D IMARIS reconstruction images (see Methods; [38,39]).

Pre-(synaptophysin) and post-synaptic (PSD95) molecular markers were used to locate the presence of pre-and postsynaptic material engulfed by GFAP-labeled astrocytes. Apotome-based (Zeiss) microscopy was used to create optical Z-stacks of GFAP-labeled astrocytes, which allowed us to perform a 3D rendering of these astrocytes to identify localization of pre- and postsynaptic material within their cytoplasm, that would indicate an engulfment process (Figure 3 and Figure 4; see also Supplementary Figures S1A,B and S2A,B).

After analyzing the relative localizations of synaptophysin, PSD95 and GFAP, we found an elevated number of pre- and postsynaptic elements inside the astrocytes after incubating slices with Aβ_1–42_ by compared to the control group (pre: Figure 4A,B,E,F; and Supplementary Figure S1A; post: Figure 4C,D,G,H; and Supplementary Figure S1B) indicating an increased synaptic engulfment had occurred.

The co-localization was also analyzed after gassing the Aβ_1–42_ pre-treated brain slices with Iso or Xe and the subsequent removal of the anaesthetics. Localization of either pre- or postsynaptic elements within GFAP-labelled astrocytes significantly decreased as compared to the Aβ_1–42_ group in both Iso and Xe treated groups (Figure 4 and Supplementary Figures S1A,B and S2A,B).

### 2.4. Both Iso and Xe Decreased MEGF10 Expression in Astrocytes either in the Presence or in the Absent of Aβ_1–42_

**Figure 5:**
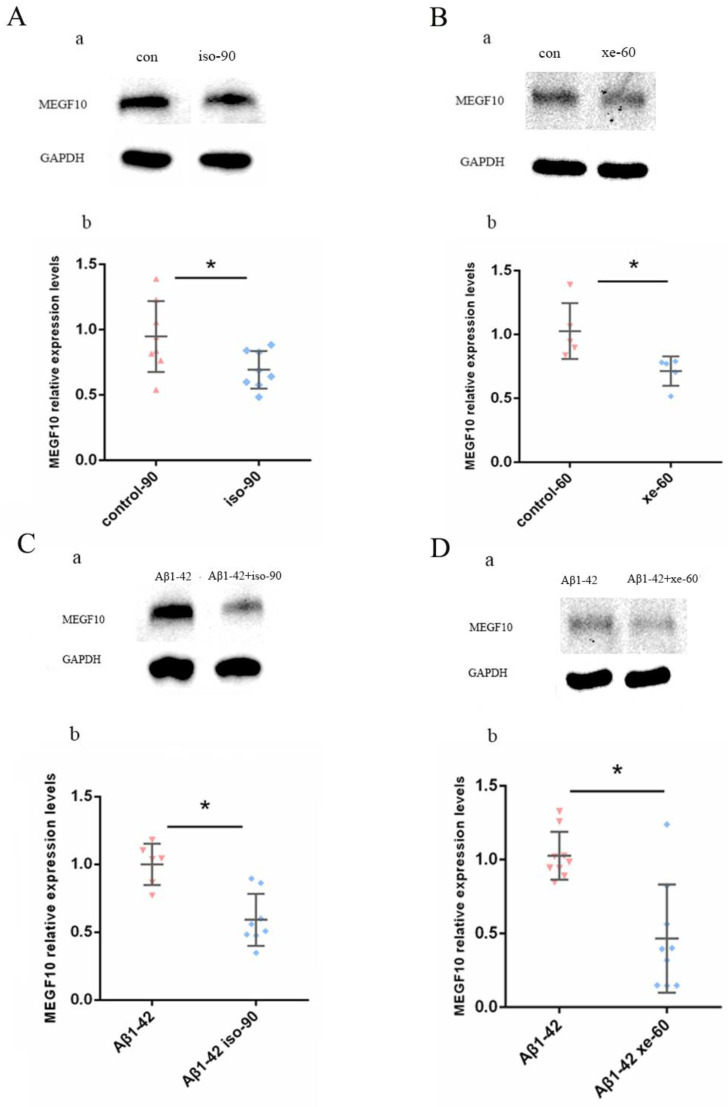
Both Iso and Xe could reduce the expression of MEGF10 on the in vitro cultured astrocytes. (**A**,**B**) show representative Western blot membranes loaded with either Iso (**Aa**) or Xe (**Ba**) treated cells. (**Ab**)—after gassed the cells with 0.37 mM Iso, the expression of MEGF10 decreased (*t*-test, * *p* = 0.0342). (**Bb**)—after the exposure to 1.9 mM Xe, the expression of MEGF10 decreased (*t*-test, * *p* = 0.0221). (**C**,**D**) both Iso and Xe decreased MEGF10 expression in astrocytes in the presence of Aβ_1–42_. Shown are representative Western blot membranes loaded with Aβ_1–42_-treated cells and combined with either Iso (**Ca**) or Xe (**Da**). (**Cb**,**Db**) are quantitative analysis showing a decrease of MEGF10 expression in astrocytes when treated in combination of Aβ_1–42_ with either Iso ((**Cb**), Mann–Whitney U test, * *p* = 0.0047) or Xe ((**Db**), *t*-test, * *p* = 0.007). GAPDH immuno-bands serves as the loading control. Error bars in all graphs indicate the means +/− SDs. Every data point in the scatter blot represents one animal. The tissues for the Western blot are from the C57BL/6 mice.

The expression of MEGF10 levels was tested in cultured astrocytes by Western blot. After exposure to 0.37 mM Iso, the expression of MEGF10 was significantly downregulated by compared to the control group (Figure 5A). The same effect was observed when the cells were gassed with 1.9 mM Xe (Figure 5B). Next, the MEGF10 protein expression in the presence of Aβ_1–42_ was tested and 90 min after exposure to 0.37 mM Iso, the expression of MEGF10 was down-regulated compared to the Aβ_1–42_ control group (Figure 5C). The same phenomenon was also observed when the cells were gassed by 1.9 mM Xe. The significant decrease was observed 60 min after Xe exposure compared to the Aβ_1–42_ control group (Figure 5D).

### 2.5. AAV-Induced Knock-Down of MEGF10 Reduced Pre- and Postsynaptic Components inside Astrocytes 

**Figure 6:**
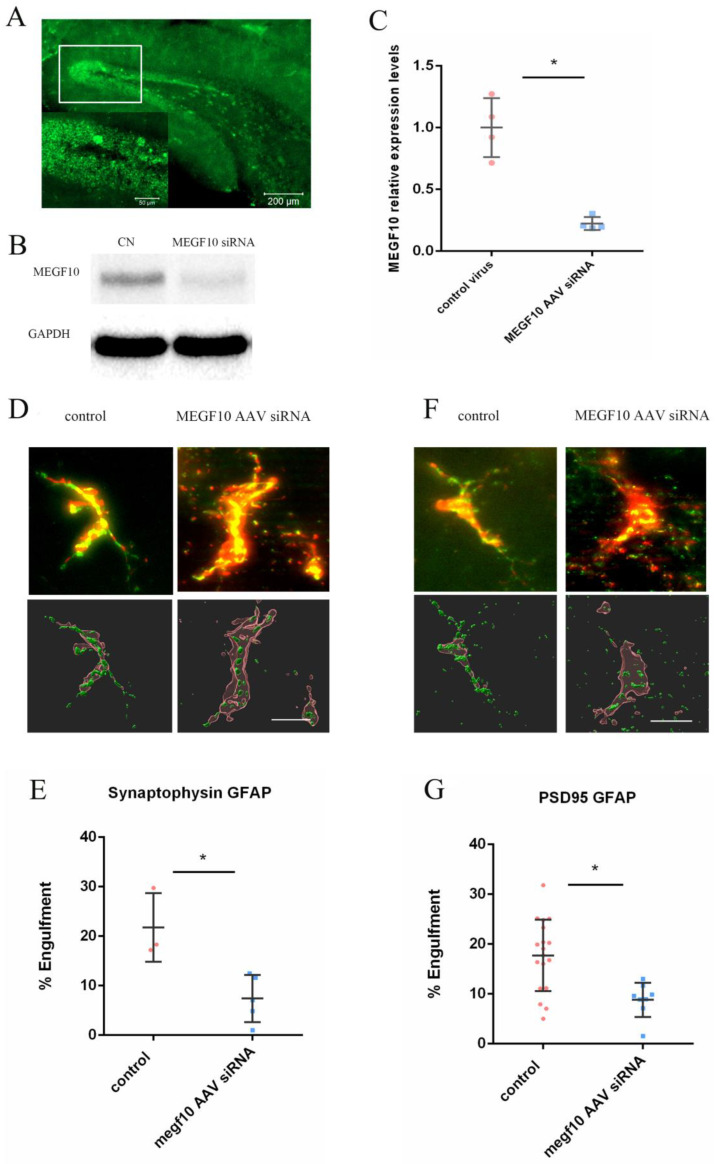
AAV-MEGF10 siRNA knocked down MEGF10 expression in astrocytes of the dentate gyrus. (**A**) shows the expression of enhanced GFP in the dentate gyrus, indicative of neurons infected by the control virus or AAV-siRNA MEGF10. (**B**) shows representative Western blot membrane of the AAV injected brain slices. (**C**), the AAV-siRNA MEGF10 significantly reduced the expression level of MEGF10 in hippocampi as compared to the control virus (*t*-test * *p* < 0.05). (**D**–**G**) show that AAV-induced knock-down of MEGF10 reduced the pre- (synaptophysin) and postsynaptic (PSD95) elements inside astrocytes (% of engulfment [38,39], Mann–Whitney U test, pre: * p = 0.0357. post: * p = 0.0062). (**D**,**F**) (top panels) show apotome fluorescent images, where red (GFAP) and green synaptic elements (**D** = synaptophysin; **F** = PSD95) are shown. The yellow colour in these panels represent the merge of GFAP and their respective synaptic elements within astrocyte. Bottom panels in (**D**,**F**) represent the same cells but rendered into 3D using IMARIS software. Scale Bars = 10 μm. Error bars in all graphs indicate the means +/− SDs. Every data point in the scatter blot represents one animal. The tissues for the Western blot are from the AAV transfected C57BL/6 mice. The images are from the AAV transfected C57BL/6 mice.

**Figure 7:**
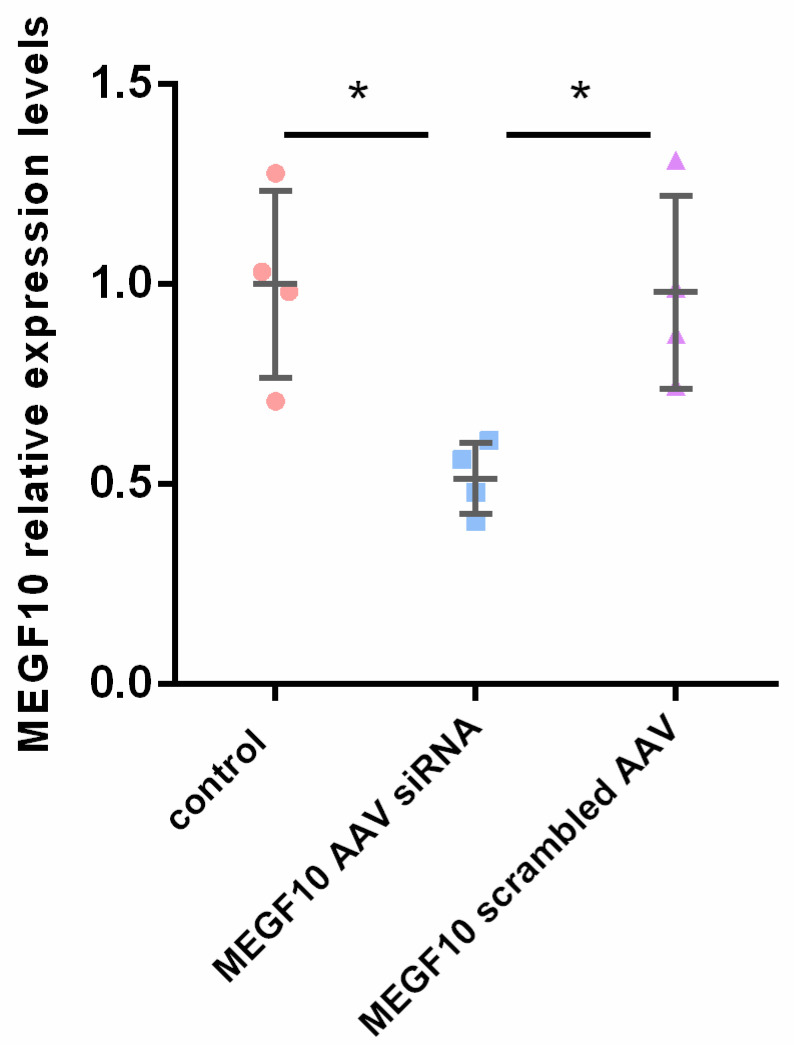
AAV-MEGF10 siRNA knocked down MEGF10 expression in cultured hippocampal astrocytes the AAV-siRNA MEGF10 reduced the expression level of MEGF10 in cultured astrocytes as compared to control (non-infected astrocytes; * *p* = 0.0189, one way ANOVA test) and scrambled (control) MEGF10 AAV (* *p* = 0.0234, one way ANOVA test).

The AAV type 9 was applied to deliver the MEGF10 siRNA to the dentate gyrus of the hippocampus to knock down the expression of MEGF10. AAV-MEGF10 siRNA significantly reduced the protein level of MEGF10 in the hippocampus as compared to the application of the control virus (Figure 6A–C). After MEGF10 knockdown, the quantification of pre-and postsynaptic components revealed a significant reduction of these synaptic elements inside the astrocytes in the dentate gyrus (Figure 6D–G). As a positive control, we found that the AAV-MEGF10 siRNA reduced MEGF10 protein also when applied to cultured astrocytes (see Figure 7).

## 3. Discussion

In this in vitro study performed in hippocampal brain slices, we found that (1) Iso and Xe reduced DSD, whereas the reduction caused by Xe was reversible; (2) Aβ_1–42_ induced a reduction of DSD in the dentate gyrus region of the hippocampus, while the combined application with either Iso or Xe reversed this spine attenuation 90/60 min after anaesthetics have been removed; (3) both anaesthetics reversed the elevation of pre- and postsynaptic material inside astrocytes caused by Aβ_1–42_; (4) both Iso and Xe downregulated MEGF10 expression in astrocytes; and (5) AAV-induced knock-down of MEGF10 reduced the occurrence of pre- and postsynaptic material inside astrocytes. 

Consistent with previous studies [40], we found that Iso exposure decreased DSD in the dentate gyrus. By contrast to Iso, the DSD reduction caused by Xe reversed after 60 min removal. This is in line with a previous study showing that Xe (1.9 mM) reversibly attenuated hippocampal LTP induction [27], suggesting that Xe could influence hippocampal synaptic plasticity, and hence also dendritic spine dynamics. It is important that the design of in vitro studies closely reflects physiological/pathophysiological conditions. One crucial parameter for this goal is the application of clinically relevant concentrations of the respective anaesthetic to generate data with high significant impact. To assure a sufficient oxygen supply and to avoid a change in pH during the slice experiments, the maximum Xe concentration which can be applied is limited to 65% or 1.9 mM. In the present study, we assessed the equipotency for Xe and Iso according to the ability to interfere with LTP. In that case, 0.37 mM Iso (≈1.3–1.4 MAC for mouse) [41] and 1.9 mM Xe (=1 MAC human or 0.4 MAC in mouse; see also [42,42]). The dose of Iso in this experiment was three-fold more than Xe. Therefore, the effect of Iso on dendritic spine dynamics may be more intensive than Xe, which might explain Xe’s reversibility regarding the effect on dendritic spines. Considering that the regular time window for one operation is 90 min, Iso was employed for a period of 90 min. Xe was applied with a shorter duration, since, as previously reported, Xe [43] combines a lower (0.115 vs. 0.59) blood gas partition coefficient than Iso [44]. Considering the blood gas partition coefficient and clinical application, Xe was employed with a duration of 60 min in this study, However, we do acknowledge that the anaesthetic and environmental conditions used, our acute ex vivo mouse brain slice preparation may have limited translation to the clinical setting.

Pharmacologically, the two anaesthetics are representing two different mechanisms of action (MOA): whereas Iso binds preferentially to GABAA receptors, Xe is a low potent antagonist at NMDA and AMPA receptors with no effects at all on GABAA receptors [45,46,47,48]. Thus, the presented results revealed that by contrast to Iso, the effect of Xe on DSD was reversible which may be due to the different exposure times and MOA.

Our results demonstrated that Aβ_1–42_ decreases DSD in the dentate gyrus region of the hippocampus, which is consistent with previous studies in hippocampal pyramidal neurons and in hippocampal CA1 region [49,50]. Furthermore, Aβ_1–42_ decreases excitatory synaptic inputs and induces the loss of synaptic contacts [51,52], showing synaptotoxic effects. It has been reported that LTP was associated with the formation of new synapses [53,54] and Aβ_1–42_ antagonizes hippocampal LTP [49,55], suggesting that Aβ_1–42_ inhibits the process of generating new synapses. Previous literature [56,57] revealed that Aβ_1–42_ elevates neuronal Ca^2+^-influx, increases AMPA receptor-mediated synaptic transmission [58] and down regulates GABAA receptors [59,60] hence producing an overall elevated network excitability. Since overexcitement in the hippocampus resulted in excitotoxic processes and loss of spines [61], it might be conceivable that for compensation, neurons reduce excitatory connections by reducing spines or internalize NMDA or AMPA receptors thereby preventing overexcitation [62,63].

As a major finding, this study demonstrated that both Iso and Xe reversed the effect of Aβ_1–42_ on DSD and astrocyte-dependent synaptic engulfment only after a 90/60 min removal but not immediately after the exposure with anaesthetics. Due to their MOA, both anaesthetics reduce neuronal excitability which might counteract the Aβ_1–42_-induced neuronal overexcitation. Antagonizing AMPA and NMDA receptors and enhancing GABAA receptor activity by Xe and Iso, respectively, might be the potential mechanisms underlying the reversal effects of both anaesthetics. Interestingly, this reversibility did not occur immediately after the removal of the anaesthetics, but first after an additional time period. The reason for this phenomenon might be due to the fact that the reversal of an already established neurotoxic process requires a longer time period than the prevention of a neurotoxic mechanism. Furthermore, our aim, though, did not focus on long-term prophylaxis against AD or AD progression. Rather, we intended to characterize possible protective effects of anaesthetics on an experimentally induced state that mimics a manifest Aβ-derived AD pathology, resembling the clinical situation, e.g., patients suffering from AD and requiring anaesthesia for surgery. Of course, the post-translational processing of Aβand aggregation course during the long-term development of AD is not achievable by a 90 min application of Aβ_1–42_.

Dendritic spines are highly dynamic structures or protrusions [64] and the decrease of DSD might be the consequence of either less formation or more elimination. Aβ_1–42_ elevated pre-and postsynaptic material inside astrocytes, supporting the idea that an increase in synapse elimination may contribute to DSD reduction. MEGF10, a transmembrane protein existing on the surface of macrophages and astrocytes [32], has been shown to be responsible for astrocyte-dependent synapses elimination [30]. In the presence of Aβ_1–42_ the expression of MEGF10 in astrocytes was down-regulated after the application of either Iso or Xe, implying that an astrocytic-dependent synapse elimination was reduced. Analyzing the localization showed that Iso and Xe inhibited phagocytosis of synapses in the presence of Aβ_1–42_, indicating that a reduced clearance might be one reason for the reversal of the reduced DSD. GABA and glutamate receptors have been reported to be expressed by astrocytes [65,66,67], implying that Iso and Xe also interact with those receptors on astrocytes. The astrocytic GABA receptor may be involved in extracellular ion homeostasis and pH regulation, while functional glutamate receptors expressed on astrocytes can modulate other channels and receptors of the same cell [68,69]. In addition, AMPA receptors are involved in the modulation of gene expression [69]. Together, those findings may provide a potential explanation why Iso and Xe could influence the expression of MEGF10. The fact that when MEGF10 was down regulated via AAV-siRNA, co-localization of pre- and postsynaptic material inside astrocytes was strongly reduced, provides further evidence that MEGF10 was indispensable to spine phagocytosis. Our results suggest that Iso and Xe reversed the Aβ_1–42_-induced enhancement of synaptic elimination presumably through the MEGF10 pathway.

In conclusion, the findings in the present study demonstrated that Iso and Xe reduced the DSD per se, whereas the effect of Xe was reversible, implying neuroprotection in the presence of Xe. Iso and Xe counteract the Aβ_1–42_-induced DSD reduction presumably through lowering synapses elimination via the MEGF10 pathway. These results provide evidence that both anaesthetics do not trigger or promote Aβ-derived pathogenesis in the progression of Alzheimer’s disease (AD).

## 4. Materials and Methods

### 4.1. Animals and Brain Slices Producing

Male C57BL/6 mice were obtained from the Charles River Lab (Germany)) (8 weeks) and Two female C57BL/6 mice and one male C57BL/6 mice were bred for puppies. Green Florescent Protein Male (GFP-M) mice (Thy1-eGFP) [70] were purchased from the Jackson Laboratory (Stock No: 007788, ME, USA), and were interbred with female C57BL/6 to generate. The genotyping of the new generation was performed 3 weeks postnatal, the tissues from the ears were sent to the genotyping lab of Charles River laboratory (Germany). The mice were decapitated after being anaesthetized with Iso. An additional Western blot experiment was performed to verify that short Iso pre-exposure for anaesthetizing before decapitation does not affect MEGF10 expression (see Supplementary Figure S3). Brains were removed quickly and sagittal slices were prepared in the ice cold, carbogen (95% O_2_, 5% CO_2_) saturated cutting solution (125 mM NaCl, 2.5 mM KCl, 25 mM NaHCO_3_, 0.5 mM CaCl_2_, 6 mM MgCl_2_, 25 mM D-glucose, and 1.25 mM NaH_2_PO_4_) into 100 µm by using a vibratome (Leica VT1000s, Leica Biosystems, Wetzlar, Germany). The slices were incubated in the carbogen saturated artificial cerebrospinal fluid (125 mM NaCl, 2.5 mM KCl, 25 mM NaHCO_3_, 2 mM CaCl_2_, 1 mM MgCl_2_, 25 mM D-glucose, and 1.25 mM NaH_2_PO_4_) at 35 °C for 30 min, subsequently in the room temperature for another 60 min. All Chemicals were sourced from Sigma-Aldrich, Taufkirchen, Germany. Experimental protocols were approved by the ethical committee on animal care and use of the government of Bavaria, Munich, Germany.

### 4.2. In Vitro Astrocyte Culture

C57BL/6 pups postnatal (P) day 0 to 4 (P0–P4) were decapitated after being anaesthetized with Iso, and the brains were removed quickly. Under the dissecting microscope, the hippocampus was dissected carefully in the ice-cold Hank’ss Buffer (Sigma-Aldrich, USA, Cat No. H6648) and was incised into 1 mm pieces. The tissue pieces were digested by 0.125% trypsin at 37 °C for 30 min and washed twice by Hank’s solution after digestion. Cells were seeded in the flask containing DMEM-F12 medium (Sigma-Aldrich, USA, Cat No. D9785). The medium was changed every 2 days. The cells were shaken on a shaker for 12 h for eliminating the microglia and Oligodendrocytes 7 days later. The supernatant containing the microglia was discarded. Astrocytes adhered on the bottom of the flask was washed with Hank’s Buffered SS (HBSS, Sigma-Aldrich, USA, Cat No. H6648) twice. The in vitro cultured astrocytes were regarded as mature after being cultured for 14 days.

### 4.3. Amyloid Beta Application and Exposure to Anaesthetics

**Figure 8:**
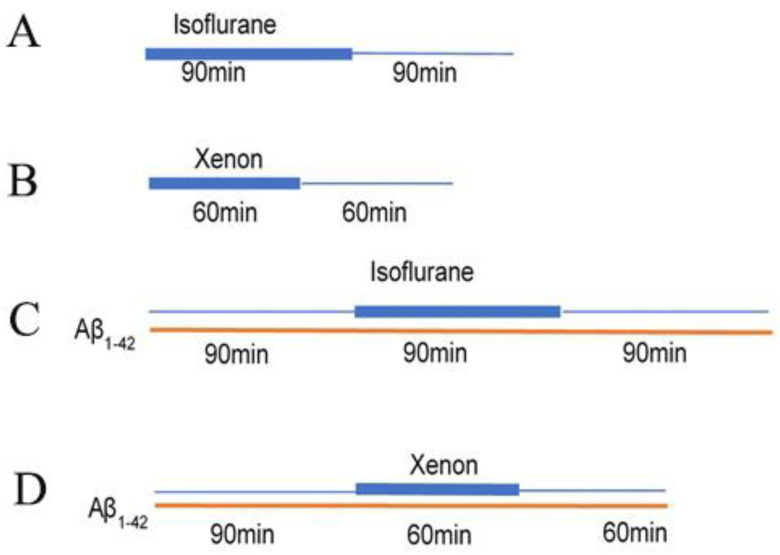
The timeline and workflow of Amyloid Beta application and exposure to Anaesthetics. (**A**) timeline of Iso exposure. The thicker blue line represents Iso gassing and thinner blue line represents Iso washing out. (**B**) timeline of Xe exposure. The thicker blue line represents Xe gassing and thinner blue line represents Xe washing out. (**C**) timeline and workflow of Amyloid Beta application and exposure to Iso. The thin orange line represents the application of Aβ_1–42_. The first thinner blue line represents gassing without Iso. The thicker blue line represents Iso gassing and the second thinner blue line represents Iso washing out. (**D**) timeline and workflow of Amyloid Beta application and exposure to Xe. The thin orange line represents the application of Aβ_1–42_. The first thinner blue line represents gassing without Xe. The thicker blue line represents Xe gassing and the second thinner blue line represents Xe washing out.

The Aβ_1–42_ was prepared as described previously [49]. Aβ_1–42_ was dissolved into a concentration of 100 µM by DMSO (ThermoFisher, Kandel, Germany, Cat No. 12345) with the aid of ultrasonic water bath. The Aβ_1–42_ was further diluted with artificial cerebrospinal fluid (aCSF; ingredients see above) into a concentration of 50 nM for the following incubation. Prepared slices and in vitro cultured astrocytes were incubated with Aβ_1–42_ (50 nM) in carbogen saturated aCSF for 90 min. The Aβ_1–42_ existing aCSF was aerated with the following different mix gases, respectively. 65% Xe (1.9 mM in aCSF)/30% O_2_/5% CO_2_ + 95% O_2_/5% CO_2_ for 60 min, Iso (0.37 mM in aCSF; gas-chromatographic measurements were described as before [41]) + 95% O_2_/5% CO_2_ for 90 min. To assure a sufficient oxygen supply and to avoid a change in pH during the slice experiments, the maximum Xe concentration which can be applied is limited to 65% [42]. Since this Xe concentration reversibly inhibits LTP and LTP is associated with the formation of new synapses and spines [53,71], we referenced this concentration to achieve the respective equipotency to Iso inhibiting LTP. According to Haseneder et al. [41], the transition from concentrations of Iso not blocking LTP to concentrations blocking LTP occurred at 0.37 mM. Thus, in this study we applied Iso and Xe at concentrations of 1% and 65%, respectively. Due to these constraints, we performed experiments under human MAC (Minimum alveolar concentration) equipotent concentrations (i.e., Iso 1% [41]) and Xe 65% [72]). After exposure to those gases, the slices and cells were gassed with carbogen for another 60/90 min for the anaesthetics washing out. The control groups were gassed only by carbogen. A summary of the timeline and workflow of the above treatments is summarized in Figure 8. Iso, Xe and carbogen gases were supplied by Linde Gas (Unterschleissheim, Germany).

### 4.4. Analysis of Dendritic Spine Density

After different treatments, the 100 µm GFP-M brain slices were fixed with 4% paraformaldehyde (PFA; Sigma-Aldrich, USA) in Phosphate buffered saline ph7.4 (PBS, Sigma-Aldrich, USA) overnight. After being washed with PBS 3 times, the brain slices were incubated with PBS + 0.5%Triton-X (Sigma-Aldrich, USA) + 10% normal goat serum (Sigma-Aldrich, USA) for 2 h at room temperature. Subsequently, for further enhancement of the GFP signal, we incubated brain slices with Rabbit anti-GFP directly conjugated to Alex Fluor 488 (ThermoFisher, Kandel, Germany, Cat No. 40943) diluted 1:200 in PBS for 2 h at room temperature in the dark. The brain slices were washed in PBS and mounted with Dako Fluorescence Mounting Medium (Dako North America Inc, CA, USA).

The Zeiss confocal microscope was used to image the dendron of the neuron in the dentate gyrus. The ZEN Black software was used for imaging acquisition. All 13-layer were detected with 0.61 mm interval in Z-stack. The final images were generated in a constant frame size of 512 × 256 pixels. 15 dendron are taken from one hippocampus. After took all the pictures of the dendron, the black ZEN software was used to measure the dendritic spines density. the tool ‘open Bezier’ was used to measure the length of the dendron, the tool ‘circle’ was used to circle the spines in different Z-stack slice.

Every data point in the scatter plots represents the mean spine density from 8–10 dendrites per one animal (n = 8–13). Dendrites that were 15 µm away from their respective cell’s soma were chosen for these analyses.

### 4.5. AAV Mediated MEGF10 Gen Knock Down

We used the AAV 9 (Figure 8) to deliver the small interfering RNA (siRNA) to the hippocampus to suppress the expression of MEGF10. The target sequence of the siRNA is: 

805-CCTGAGGGTCGCTTTGGAAAGAACTGTTC2710-

GCAGACTATACCATCGCAGAAACCCTGCC3136- 

TGCGGCTACGTGGAGATGAAGTCGCCGGC3215- 

GGAATGTCTATGAAGTCGAACCTACAGTG. 

MEGF10 siRNA AAV9 (4.72 × 1012 genome copies per mL) and control siRNA AAV9, which refers to scrambled AAV siRNA (3.56 × 1012 genome copies per mL) were produced by abm-Canada (https://www.abmgood.com/; accessed on 1 September 2017). In brief, abm-Canada cloned in MEGF10 siRNA and MEGF10 scrambled siRNA into AAV viral plasmid (Figure 8A,B, respectively). Next a 3 plasmid transfection into HEK-293 cells, according to the method of Griger et al., 2006 was done [73]; for example, MEGF10 siRNA AAV plasmid (Figure 8A), pXX9 plasmid [coding for the Adenovirus helper genes], and pRepCap plamid cosing for AAV capsids]). Transfected HEK293 cells were then homogenized and AAVector particles were purified and supplied by abm Canada at the above-mentioned vector genomes per milliliter. 

Stereotaxic injection of these AAV vector particles was performed as before [74]. Briefly, 0.5 μL of AAV was delivered bilaterally to the dentate gyrus (1.8 mm posterior to bregma, 1.2 mm lateral from midline and 1.65 mm dorsoventral from dura) of 8-week-old adult mice over a 10 min period. The micropipette was left in the site for additional 10 min. After 3 weeks of recovery, the mice were sacrificed. Cultured astrocytes were incubated for 2 days with either AAV-Megf10, or control siRNA AAV9. Cell lysates from infected cells were made and probed for Megf10 protein expression normalized to the GAPDH loading control protein as detailed in below in Section 4.6. 

**Figure 9:**
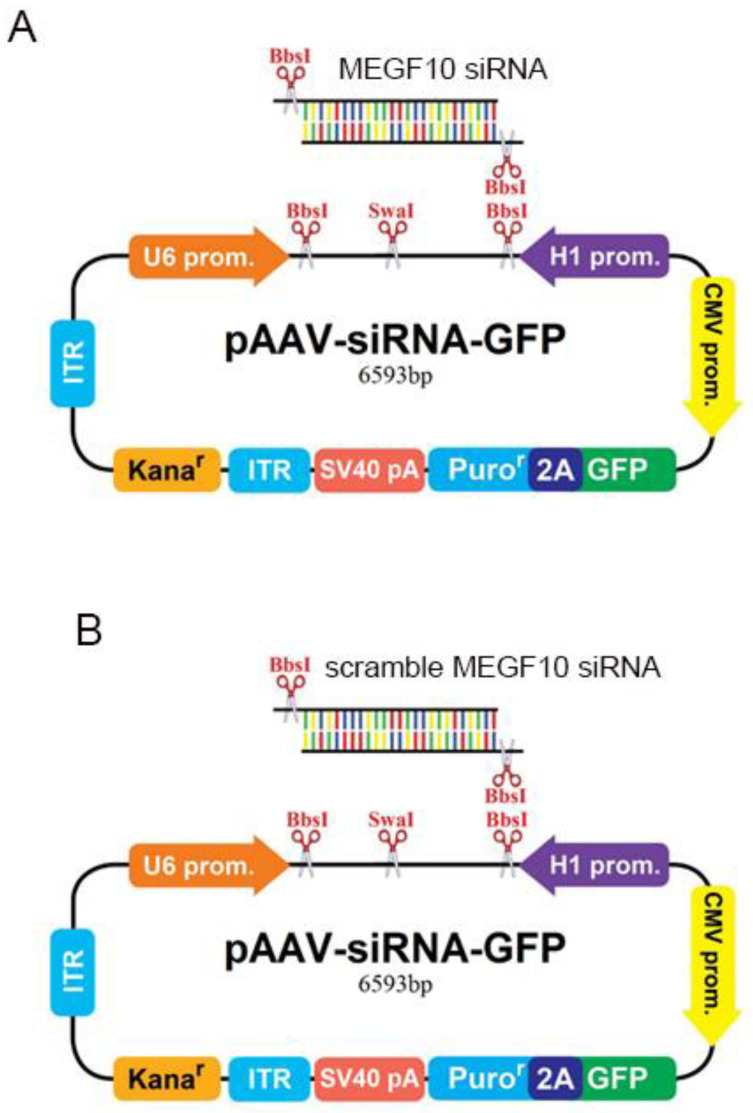
(**A**) Shows the schematic map of the pAAV-MEGF10 siRNA-GFP viral construct used. (**B**) Shows the schematic map of the pAAV-scramble MEGF10 siRNA-GFP viral construct used. The company abm-Canada did the cloning of MEGF10-siRNA AAV9 and MEGF10 scrambled siRNA AAV9. These schematics are downloaded from the abm’s web site (https://www.abmgood.com/, accessed on 1 January 2020).

Maps of the siRNA AAVs (obtained from the company abm Canada) is displayed in Figure 9. 

### 4.6. Western Blot

After treatment, the in vitro cultured astrocytes were harvested by using the lysis buffer (RIPA lysis buffer (complete), PMSF, Pepstatin, Sigma-Aldrich, Taufkirchen, Germany). The hippocampi of the slices were separated and homogenized in the ice-cold lysis buffer (RIPA lysis buffer (50× complete), 100× PMSF, Pepstatin, Sigma-Aldrich, Taufkirchen, Germany). The quantification of the proteins was determined by a bicinchoninic acid protein assay kit. For Western blotting, a total of 20 μL of the sample containing 30 μg protein was loaded per lane and the proteins were electrophoretically separated on an SDS-PAGE gel. The protein bands were transferred to a polyvinylidene fluoride membrane. After being blocked with Rotiblock (Biorad, South Granville, Australia) for 1 h, the membrane was incubated with Anti-MEGF10 antibody (1:500, Sigma-Aldrich, Taufkirchen, Germany, Cat No. ABC10) and anti-GAPDH (1:10,000, ThermoFisher, Kandel, Germany, Cat No. MA5-15738) 4 °C overnight. Expression of GAPDH was used as a loading control, and to assist in determining changes in MEGF10 protein expression. Subsequently incubated the membrane with horseradish peroxidase-conjugated secondary antibodies (1:10,000, CST, USA) for 2 h at room temperature. The bands were quantified by Imaging lab software.

### 4.7. Immunofluorescence and Synaptic Engulfment Analysis 

After being incubated with Aβ_1–42_ and anaesthetics, the 100 µm C57BL/6 brain slices were fixed by 4% PFA (paraformaldehyde) at room temperature overnight. After being washed with PBS, the slices were permeabilized and blocked with 0.3% Triton-X and 10% normal goat serum for 2 h. incubated with the first antibody, anti-GFAP (1:1000, chicken, Abcam, Berlin, Germany, Cat No. ab4674), anti-synaptophysin (1:200 rabbit, Abcam, Berlin, Germany, Cat No. ab14692), anti-PSD95 (1:200 rabbit, Cell Signalling, Leiden, Netherlands, Cat No. 3450), at 4 °C overnight. The slices were labeled with the second antibody, (donkey anti-chicken Alexa fluor594 1:500, donkey anti-rabbit Alexa fluor488 1:500; ThermoFisher, Kandel, Germany) at room temperature for 2 h keeping in dark on the following day. Images were taken by using Zeiss Apotome Fluorescence microscope with a plan apochrome objective (63× oil immersion NA = 1.4). Apotome Z-stacks images were rendered in 3D using IMARIS 9.5.1 (Bitplane, Zurich, Switzerland) (detailed below). Quantitative engulfment measurement was performed using the percentage of engulfment by the method of Schafer et al. [38,39]. One limitation of our study was that we did not specifically localize the intracellular content to the lysosome. One statistical “n” was referred as the average % engulfment of different astrocytes in one hippocampus. Typically, 3 to 5 astrocytes on the one hippocampus (*n* = 1) were chosen of these analyses. Form these 3 to 5 astrocytes a mean value was obtained and represented by one data point per animal. We recognized that GFAP may not label the finer processes of estrocytes, and thus does not comprise the best marker for assessing interactions with synapses. This is a limitation of this study.

### 4.8. Three Dimensional (3D) Rendering of GFAP-Stained Astrocytes Containing Synaptic Elements

For the pre- or postsynaptic material, the build in background subtraction function was used on the green channel, the function applies a Gaussian filter to performs a baseline subtraction of its surrounding background at each voxel (the term same as pixel for 3D) to identify structure from the noisy background. Then, the point of inflection in the intensity graph were selected as threshold to render the 3D object of both the astrocytes and synaptic material. For quantification of engulfed synaptic elements, individual astrocyte cells were selected for quantification. All immuno-labelled pre- or postsynaptic elements, were marked out and isolated for each cell, only the labelled synaptic material within the cell were quantified. The total sum of volume of the synaptic materials were compared to the corresponding cell’s volume to calculate the % of engulfment in each cells according to the method described by Schafer et al., in 2012 [38]. 

### 4.9. Statistical Analysis

Normality of distributions was tested using the D’Agostino & Pearson omnibus normality test and visual inspection of data histograms. If the data passed the normality test, data were presented as the mean ± SD, and a two-tailed unpaired *t*-test was performed to compare group difference. Data were presented as median (IQR) and a two-tailed Mann–Whitney U test was performed to compared group difference, if the data did not pass the normality test. *p* < 0.05 was considered to indicate a statistical difference. We selected *p* < 0.05 as the significance level. All statistical analyses were performed in GraphPad software.

## Data Availability

There is no data created so far.

## Supplementary Materials

The following supporting information can be downloaded at: https://www.mdpi.com/article/10.3390/ijms24020912/s1.
