# Correction: Shi et al. The Anaesthetics Isoflurane and Xenon Reverse the Synaptotoxic Effects of Aβ_1–42_ on Megf10-Dependent Astrocytic Synapse Elimination and Spine Density in Ex Vivo Hippocampal Brain Slices. *Int. J. Mol. Sci.* 2023, *24*, 912

**DOI:** 10.3390/ijms26188797

**Published:** 2025-09-10

**Authors:** Dai Shi, Jaime K. Y. Wong, Kaichuan Zhu, Peter G. Noakes, Gerhard Rammes

**Affiliations:** 1 Department of Anesthesiology and Intensive Care, Klinikum Rechts der Isar, Ismaningerstraße 22, 81675 Munich, Germany; shidoc8729@gmail.com; 2 School of Biomedical Sciences, The University of Queensland, St. Lucia, QLD 4072, Australia; jaimewky@gmail.com (J.K.Y.W.); p.noakes@uq.edu.au (P.G.N.); 3 German Center for Neurodegenerative Diseases, Feodor-Lynen-Straße 23, 81377 Munich, Germany; kc.zhu@siat.ac.cn; 4 Center for Neuropathology and Prion Research, Feodor-Lynen-Straße 23, 81377 Munich, Germany; 5 Queensland Brain Institute, The University of Queensland, St. Lucia, QLD 4072, Australia

In the original publication [1], inadvertent errors were present in Figures 1A and 1C. Specifically, the *control* images in both Figures 1A and 1C, as well as the *xe-60* image in Figure 1C, were duplicated unintentionally. These duplications resulted from an oversight during the final figure assembly. The correct Figure 1 is shown below. 

The authors state that the scientific conclusions are unaffected. This correction was approved by the Academic Editor. The original publication has also been updated.

## Figures and Tables

**Figure 1:**
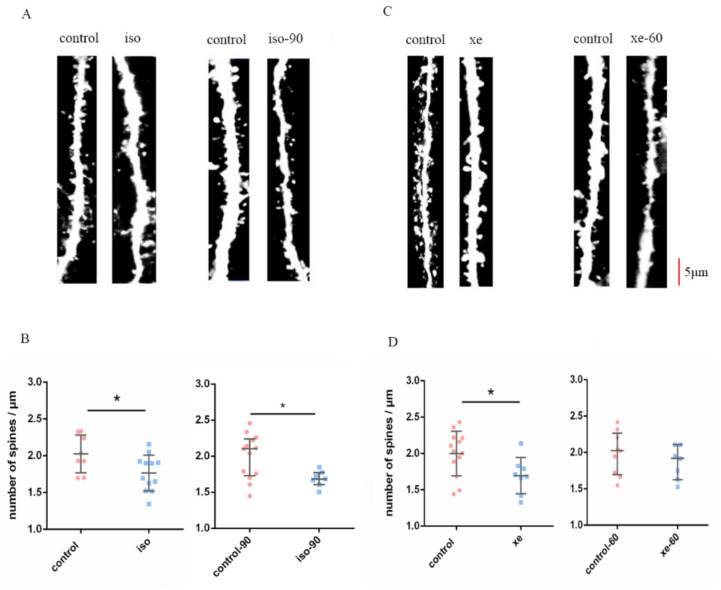
(**A**,**C**) show representative dendrites from the control group and the isoflurane (Iso)/xenon (Xe) treated groups, at treatment and post wash out (60/90 min) of gas, respectively. Scale bar = 5 μm. Dendrites were analyzed from brain slices of GFP-M mice. (**B**,**D**) left graphs show both Iso and Xe reduced the DSD in the dentate gyrus of hippocampus (*p* = 0.0201 for iso, and *p* = 0.0244 for Xe, Mann–Whitney U test). (**B**,**D**) right graphs show that after 60 mins the reduction of DSD caused by Xe was reversed (*p* = 0.3441 Mann–Whitney U test), but not for Iso after 90 mins (*p* = 0.0297, Mann–Whitney U test). Error bars in all graphs indicate the means +/− SDs. Every data point in the scatter plots represents the mean spine density from 8–10 dendrites per one animal (*n* = 8–13). Dendrites that were 15 µm away from their respective cell’s soma were chosen for these analyses. Black horizontal bars below the * indicate a significant difference (*p* < 0.05) between the groups.
