# Supplementary material for: The Anaesthetics Isoflurane and Xenon Reverse the Synaptotoxic Effects of Aβ1–42 on Megf10-Dependent Astrocytic Synapse Elimination and Spine Density in Ex Vivo Hippocampal Brain Slices
Source: Int J Mol Sci. 2023 Jan 4;24(2):912. doi: 10.3390/ijms24020912 (PMC9861496; doi:10.3390/ijms24020912)
Supplement: Supplementary file 1 [file ijms-24-00912-s001.zip › ijms-2120319-supplementary.pdf]

Figure S1A

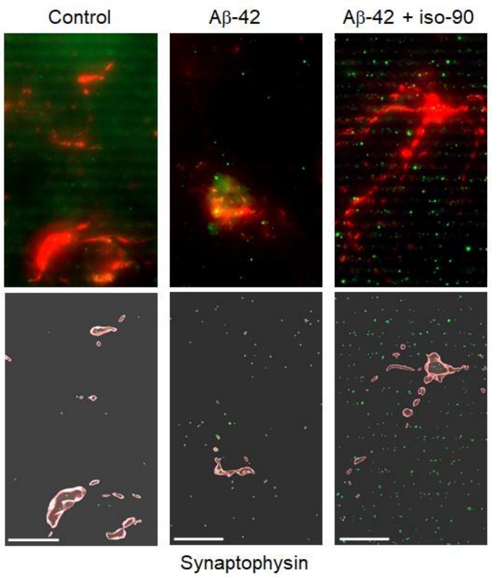

Figure S1B

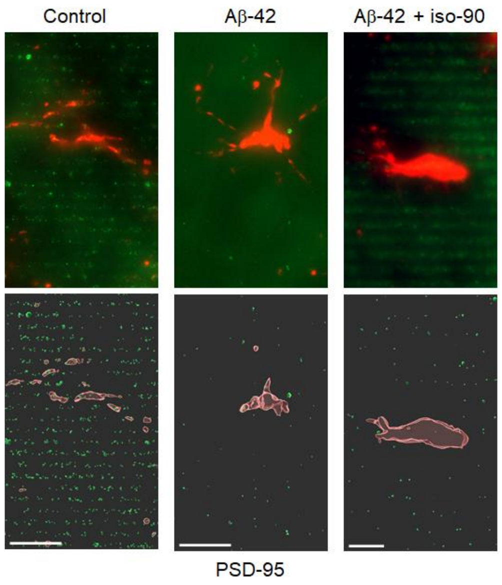

Scale bar = 10 um

Figure S1: Shows GFAP positive (red) astrocytes and locations of the pre-synaptic marker synaptophysin (green) in hippocampal slices, left untreated (control), middle Aβ-42 treated, and right Aβ-42 plus (+) isoflurane (**iso**) treated. Scale bars = 10 um

Figure S2A

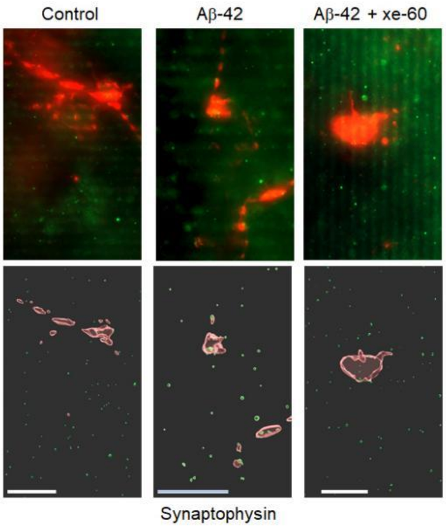

Figure S2B

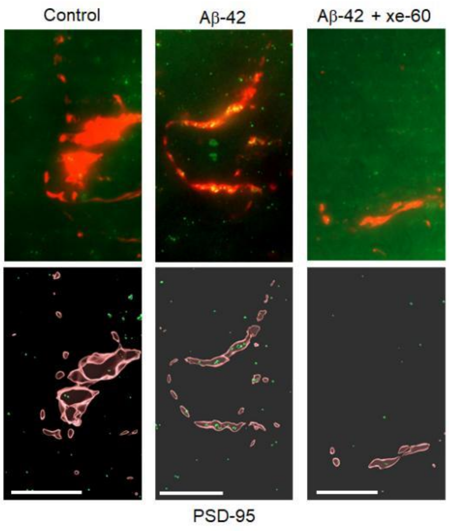

Scale bar = 10 um

Figure S2: Shows GFAP positive (red) astrocytes and locations of the post-synaptic marker PSD-95 (green) in hippocampal slices, left untreated (control), middle Aβ-42 treated, and right Aβ-42 plus (+) isoflurane (**iso**) treated. Scale bars = 10 um.

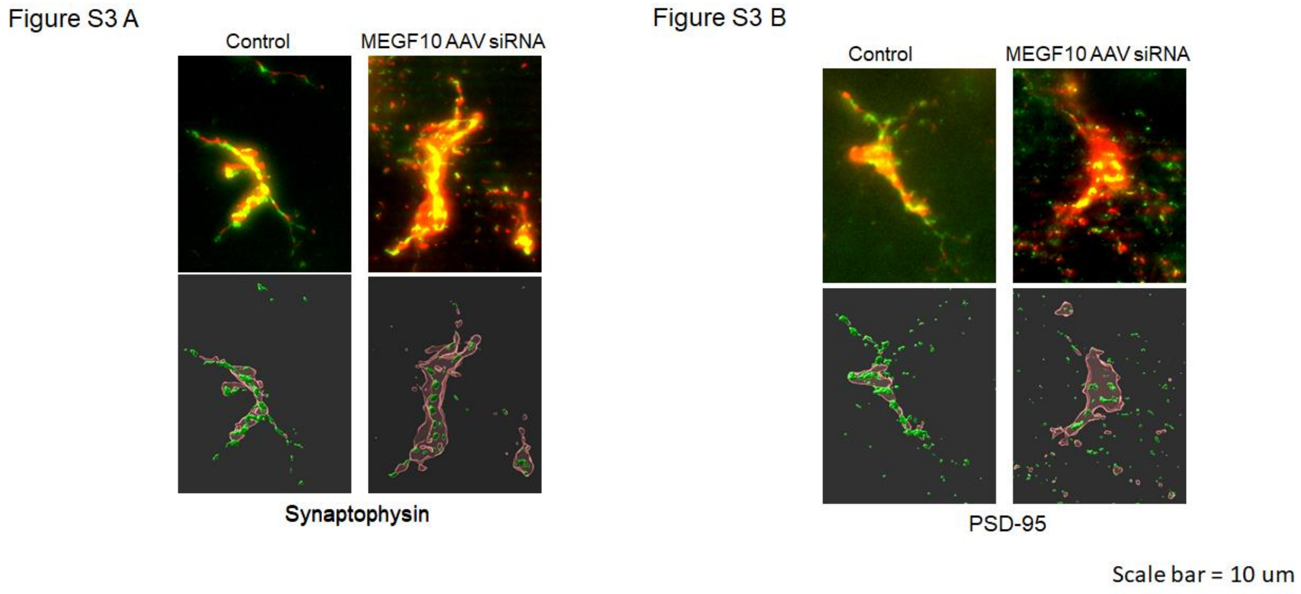

Figure S3: Shows GFAP positive (red) astrocytes and locations of the pre-synaptic marker synaptophysin (green, upper set of panels), and locations of the post-synaptic marker synaptophysin PSD-95 (green, lower set of panels), in hippocampal slices left untreated (control) and right MEGF10 knockdown (MEGF10 siRNA treated). Scale bars = 10 um

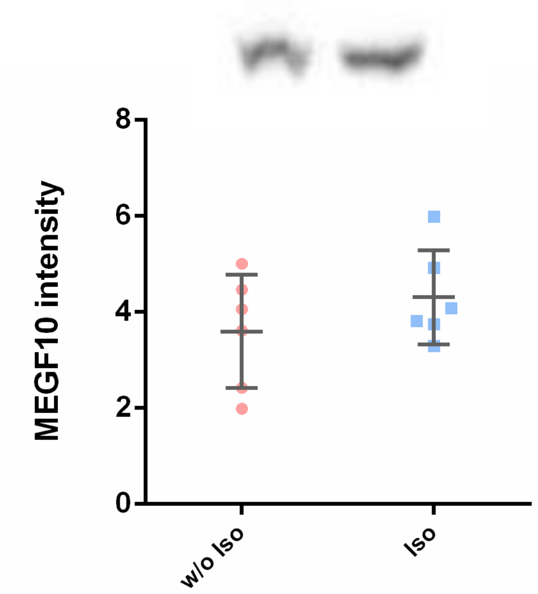

Figure S4: A short Iso exposure to anesthetize the animal previous to decapitation does not affect Megf10 levels. Analysed data from western blots (right, representative blot) show Megf10 expression without (w/o Iso) Iso anesthesia before cervical dislocation compared to a short Iso anesthesia (Iso). Megf10 levels did not change. (Mann Whitney test, P=0.4740).
